# Rethinking the Dose-Response Relationship Between Usage and Outcome in an Online Intervention for Depression: Randomized Controlled Trial

**DOI:** 10.2196/jmir.2771

**Published:** 2013-10-17

**Authors:** Liesje Donkin, Ian B Hickie, Helen Christensen, Sharon L Naismith, Bruce Neal, Nicole L Cockayne, Nick Glozier

**Affiliations:** ^1^Brain & Mind Research Institute, The University of SydneyNew South WalesAustralia; ^2^Black Dog InstitutePrince of Wales HospitalHospital Road, RandwickNew South WalesAustralia; ^3^The George Institute for Global Health, The University of SydneyPO Box M201Missenden RoadNew South WalesAustralia; ^4^Disciplines of Psychiatry and Sleep MedicineSydney Medical SchoolThe University of SydneySydneyAustralia

**Keywords:** adherence, Internet, eHealth, depression, patient compliance

## Abstract

**Background:**

There is now substantial evidence that Web-based interventions can be effective at changing behavior and successfully treating psychological disorders. However, interest in the impact of usage on intervention outcomes has only been developed recently. To date, persistence with or completion of the intervention has been the most commonly reported metric of use, but this does not adequately describe user behavior online. Analysis of alternative measures of usage and their relationship to outcome may help to understand how much of the intervention users may need to obtain a clinically significant benefit from the program.

**Objective:**

The objective of this study was to determine which usage metrics, if any, are associated with outcome in an online depression treatment trial.

**Methods:**

Cardiovascular Risk E-couch Depression Outcome (CREDO) is a randomized controlled trial evaluating an unguided Web-based program (E-couch) based on cognitive behavioral therapy and interpersonal therapy for people with depression and cardiovascular disease. In all, 280 participants in the active arm of the trial commenced the program, delivered in 12 modules containing pages of text and activities. Usage data (eg, number of log-ins, modules completed, time spent online, and activities completed) were captured automatically by the program interface. We estimated the association of these and composite metrics with the outcome of a clinically significant improvement in depression score on the Patient Health Questionnaire (PHQ-9) of ≥5 points.

**Results:**

In all, 214/280 (76.4%) participants provided outcome data at the end of the 12-week period and were included in the analysis. Of these, 94 (43.9%) participants obtained clinically significant improvement. Participants logged into the program an average of 18.7 times (SD 8.3) with most (62.1%, 133/214) completing all 12 modules. Average time spent online per log-in was 17.3 minutes (SD 10.5). Participants completed an average of 9 of 18 activities available within the program. In a multivariate regression model, only the number of activities completed per log-in was associated with a clinically significant outcome (OR 2.82, 95% CI 1.05-7.59). The final model predicted 7.4% of variance in outcome. Curve estimates indicated that significant logarithmic (*P*=.009) and linear (*P*=.002) relationships existed between activities completed per log-in and clinically significant change.

**Conclusions:**

Only one objective measure of usage was independently associated with better outcome of a Web-based intervention of known effectiveness. The 4 usage metrics retained in the final step of the regression accounted for little outcome variance. Medium level users appeared to have little additional benefit compared to low users indicating that assumptions of a linear relationship between use and outcome may be too simplistic and further models and variables need to be explored to adequately understand the relationship.

**Trial Registration:**

Australian New Zealand Clinical Trials Registry (ANZCTR): ACTRN12610000085077; http://www.anzctr.org.au/ACTRN12610000085077.aspx (Archived by WebCite at http://www.webcitation.org/6K9FQtKBn).

## Introduction

Web-based interventions for psychological conditions have been found to have a moderate to large effect size [1,2] that is comparable to face-to-face interventions [3-5]. However, in a review of Web-based interventions, the median proportion of users completing all modules in a trial was 56% [6]. Drawing on the medication literature, this level of exposure to an intervention would be considered suboptimal, but no similar models exist for Web-based interventions. Given this, it is unclear how important the degree of program usage is for outcomes in online interventions.

To date, much of the reporting of engagement is of dropout attrition [7] (the proportion of participants that do not complete the trial or provide follow-up data) or of treatment completers or persisters (those that complete the intervention). However, reporting on attrition alone does not adequately describe how the user interacts with the program nor does it inform developers of how much of the intervention needs to be completed in order for participants to obtain a benefit. An alternative way to gain these insights is to assess a measure of usage or of adherence. Usage refers to the level of activity within a program, whereas adherence refers to the degree to which the user’s activity within the program matches the pattern of activity that was intended by the program developers. For example, a user who completes all 10 modules in a program will have 100% usage on the modules’ metric of usage. However, if these modules were supposed to be completed weekly and the user only completed 6 of these on time, the user was 60% adherent on the modules’ adherence metric. Alternatively, if a user completes all 20 of the compulsory activities in a program when scheduled to do so, the user is 100% adherent. However, if the user completes these activities several times, the user’s usage statistic may be much higher. These 2 concepts provide a measure of activity within a program, with one focused on general activity (usage) and the other focused on whether this activity matches the developer’s expectations (adherence). Therefore, adherence is a specific subset of usage that has timing factors as a component of what is measured. Despite these differences, both of these variables provide important information about program engagement and provide an opportunity for researchers to understand whether it is exposure to program material or adherence that is needed to obtain a clinically significant effect.

Web-based interventions have an advantage over traditional medication trials in terms of measuring usage because there are many objective metrics readily available [8]. Such objective measures include the number of times the participant logs into the program, the number of modules completed, the number of completed activities, and broad patterns of usage, such as time spent online and the repetition of optional activities completed. Assessment may be further refined by composite measures [8], such as time spent per activity or number of modules completed per log-in. Despite the relative ease of capturing these data in online interventions, few studies report these. Even when reported, common practice is to report dropout attrition only or a singular measure of use, which inadequately describes the level of program use in these trials. Thus, little insight is gained about the impact of usage on program outcomes.

Recent articles have begun to explore the relationship between program usage and outcomes [6,8,9]. For example, based on a post hoc median split of website activity (calculated as number of log-ins multiplied by duration in minutes per log-in), high users of an Internet program aimed at smoking cessation were more likely to quit and remain continually abstinent than low users [10]. The same has been found in eating disorders in which increased completion of program components and tasks in online interventions has been found to predict better outcomes [9,11,12]. Likewise, greater improvements in anxiety and depression were seen as individuals worked through increasing numbers of modules on an online cognitive behavior therapy (CBT) program [13]. Finally, better engagement online has been found to positively influence the consumption of fruit and vegetables [8]. Such analyses indicate that the dose of the behavioral intervention appears to influence outcome [10,13-15].

A recent systematic review of Web-based interventions showed that several potential usage metrics (number of log-ins, self-reported activity completions, and time spent online) were not consistently associated with outcome for Web-based intervention for psychological disorders [16]. Only the relationship between proportion of modules completed and outcome appear to be consistent. The assumption behind these approaches is that there is a linear relationship between outcome and exposure to content. However, the relationship between dose-response may not be linear, but rather curvilinear (eg, reaches a saturation point where no further benefit is obtained). Likewise, the association may be modified by sociodemographic factors [17] or psychological traits [18,19].

The inability to consistently detect a dose-response relationship may be influenced by the usage metric utilized. Because most studies only report 1 or 2 such usage metrics and rarely examine the relationship between these metrics, little is known about the relative contribution of the different metrics or the relationship of these to outcomes. Previous attempts have been made to define the measurement of usage, most often in the form of adherence [20,21], by producing combined measures of engagement [8] and to standardize reporting of this [22,23], but variations in reporting continue to exist in the literature. These variations in reporting may be because of a lack of consensus about the relationship between usage and outcome or the best way to measure usage, leaving researchers confused. Given this, this study aims to evaluate the role of several different usage metrics and combinations of these on the outcome of a randomized controlled trial (RCT) of an online depression treatment trial. Furthermore, this study seeks to determine which of these, if any, are more important in predicting and explaining a clinically significant change. It was hypothesized that usage would be associated with outcome and that modules completed would have the strongest relationship with outcome, consistent with the systematic review by Donkin et al [16].

## Methods

### Overview

Cardiovascular Risk E-couch Depression Outcome (CREDO) is a randomized, double-blind, parallel, attention-controlled, Internet-delivered trial targeting depressive symptoms in those with risk factors for or diagnosis of cardiovascular disease (CVD). The method and primary results of CREDO have been published elsewhere [24,25]. This study is a secondary analysis of the usage of the intervention.

### Participants

Trial participants were recruited from the 45 and Up Study [26], a longitudinal study of health and aging in New South Wales, Australia. Potential participants were invited to participate in the CREDO trial if they were aged between 45 and 75 years, provided a valid email address, self-reported significant risk factors for or a history of CVD, and screened positive for at least moderate psychological distress on the Kessler Psychological Distress 10 scale (K10) [27,28] during the 45 and Up Study baseline data collection. Potential participants underwent a further screening process for trial inclusion to ensure a current level of depressive symptoms. Once identified as being suitable for the trial, participants were randomized either into the intervention arm using E-couch, an Internet cognitive behavior therapy (iCBT) intervention, or to HealthWatch, an online attention control. Both E-couch and its predecessor, MoodGYM [29,30], have been shown to be effective in improving symptoms of depression [31]. To determine the effect of E-couch usage on outcome, only those participants in this arm who completed the outcome measure at 3 months were included in this analysis.

### Intervention

E-couch is an iCBT program containing psychoeducation about depression with components of CBT, interpersonal psychotherapy (IPT), applied relaxation, and physical activity. In its open access format [32], E-couch allows users to choose which aspects of treatment they wish to engage with in the form of choosing their own toolbox. For the purpose of CREDO, the program was restructured linearly so that it contained 12 modules that required users to work through each module sequentially rather than being able to choose which section they wanted to engage with.

Activities were spread throughout E-couch. The CBT component had 12 activities, IPT had 4 activities, and the exercise component had 2 activities. The relaxation component contained a recording of relaxation exercises, but because this did not require participants to enter anything into the program, it was not included as an activity in this analysis. See Multimedia Appendices 1-4 for screenshots of exercise examples. Users were sent an email when their module opened and a reminder email again 3 to 4 days later if the module had not yet been completed. If they still had not completed the module 1 week after it was opened, they received a reminder phone call prompting their return to the site.

### Outcome Measure

The primary outcome measure of the study was the 9-item Primary Health Questionnaire (PHQ-9) [33], a widely used self-report tool designed for the assessment of depressive symptoms in community samples. Items are scored on a scale of 0 to 3 and are provided with a summary score ranging from 0 to 27. The PHQ-9 has shown to have sufficient sensitivity and specificity for major depressive disorder [29,33] and to be an indicator of minimal clinically important change for individuals [34]. For this analysis, the standard definition of a clinically significant improvement of a reduction of 5 points in PHQ-9 score [33] was used as the outcome measure. This was utilized in favor of a continuous measure because it was considered to be the most clinically meaningful.

### Usage Metrics

#### Overview

A number of measures were used to assess usage of the intervention as recorded objectively by the program and did not rely on participant self-report.

#### Proportion of Modules Completed

The proportion of the 12 possible E-couch modules that the individual completed was recorded. A complete module consisted of the user clicking through each page of the module until they had viewed all pages. No time limit or activity level was required to complete the module other than what was required to click through the module’s pages.

#### Proportion of Activities Completed

Data were captured for each type of activity section (ie, cognitive activities, relationship activities, and physical activities) and overall activity completion. A total of 18 different activities were available for completion in the program and were spread throughout the modules. To complete more activities, users needed to complete more modules. In order for an activity to be counted as completed, the individual had to have engaged with the task in some way (eg, provided text or worked through the activity by clicking on the required sections).

#### Number of Program Log-Ins

The number of times the participant logged into the program over the course of the 12-week period was recorded. Participants were expected to complete 1 log-in per week; therefore, they were expected to have logged into the program on 12 occasions. All modules could theoretically be completed in 1 log-in at week 12 (a module being made available each week for 12 weeks). Participants were able to log in as many times as they wished per module, allowing this metric to range from 1 to an unknown limit imposed by the study duration and participants’ availability.

#### Total Number of Activities Completed

Users were able to complete each activity as many times as they wanted and were not limited to 18 activities. Given this, the total number of activities completed was collected.

#### Total Time Spent in the Program

The total time spent logged into the program each week was recorded. The program continued to keep time if the user did not log out; therefore, the time spent on the final page for each log-in was excluded in case of failure to log out. Average time spent online per log-in and total time in the program were used in this analysis. Average time spent online per log-in was capped at 60 minutes to reduce the impact of outliers. This impacted 1 participant; average time spent online per log-in was limited to 60 minutes from 83 minutes. No minimum average time requirement was defined for the program.

#### Average Number of Activities Completed per Log-In

This was calculated by dividing the total number of activities completed by the total number of log-ins to the program.

#### Average Number of Minutes per Log-in

This was calculated by dividing the total time in minutes spent in the program by the total number of log-ins to the program.

#### Average Number of Modules Completed per Log-in

This was calculated by dividing the number of modules completed by number of times that they logged in to the program.

#### Combined Modules-Activities Measure

An aggregated measure was calculated by adding the number of modules completed (range 0-12) with the number of compulsory activities completed (range 0-18) to give a total range of 0 to 30.

### Data Analysis

Data analyses were completed using SPSS version 20.0 (IBM Corp, Armonk, NY, USA). Data were examined for normality and where the assumptions of normality were not met, nonparametric tests were utilized. Chi-square (χ^2^), independent samples *t* tests, and Mann-Whitney *U* tests were used to determine if there were any differences between those who persisted with the study (ie, provided postintervention outcome data at week 12) and those who did not. Univariate associations of demographic variables with outcome and usage were evaluated using Spearman rank correlation (ρ) and chi-square tests. Similarly, Spearman rank correlations, independent samples *t* tests, Mann-Whitney *U* tests, and chi-square tests were used to examine the relationship between usage variables and clinically significant improvement.

A binary logistic regression model using the enter method was then completed to assess the ability of the usage variables to predict clinically significant improvement. Demographic and usage variables were included in the regression model if there was *P* value of *P*<.20 for its association with the outcome. Autocorrelations between usage variables were assessed before modeling. Where significant autocorrelations were found (considered to be a correlation of *r*>0.80), the variables were identified as a priori (ie, being more relevant) and were entered into the model.

### Ethical Approval

Written informed consent was obtained from all the participants and ethics approval for the 45 and Up Study was provided by the University of New South Wales Human Research Ethics Committee. Ethics approval for the CREDO trial was obtained from the University of Sydney Human Research Ethics Committee.

## Results

### Overview

Of the 562 participants who provided consent and met trial criteria, 280 (49.8%) were randomized into the E-couch iCBT program. Of these, 214 (76.4%) persisted with the study and provided postintervention outcome data. There were no significant differences between persisters and those who did not provide outcome data in age, sex, country of birth, marital status, or baseline depression severity (Table 1). However, those who spoke English at home were more likely to persist with the trial (OR 2.91, 95% CI 1.51-7.38). As indicated in Table 1, significant differences existed between persisters and nonpersisters on all 3 basic usage metrics.

**Table 1:** Association of demographics, baseline depression score, and basic usage metrics with study persistence.

Variable	Persisters (n=214)	Nonpersisters (n=66)	OR (95% CI)	*t* (df)	*P* value
Age (years), mean (SD)	57.39 (6.5)	57.68 (7.1)		0.31 (278)	.75
Depression score (PHQ-9), mean (SD)	12.13 (3.5)	11.27 (3.0)		–1.81 (278)	.07
Sex (female), n (%)	133 (62.1)	40 (60.6)	1.07 (0.61-1.88)		
Speak English at home, n (%)	203 (94.9)	57 (86.4)	2.91 (1.15-7.38)		
Marital status (partnered), n (%)	151 (70.6)	53 (80.3)	0.59 (0.30-1.15)		
Highest qualification (postschool), n (%)	157 (73.4)	47 (71.2)	1.11 (0.60-2.06)		
Born in Australia, n (%)	158 (73.8)	53 (80.3)	0.69 (0.35-1.36)		
Number of modules completed (range 0-12), mean (SD)	12 (3.8)	3.21 (2.1)		–21.05 (196)	<.001
Number of activities completed (range 0-44), mean (SD)	11.9 (9.1)	1.6 (3.0)		–14.18 (276)	<.001
Number of log-ins (range 1-65), mean (SD)	18.72 (8.3)	7.18 (4.7)		–14.25 (196)	<.001

### Intervention Usage of Those Who Persisted With the Study

Of the 214 participants, 62.1% (133/214) completed all 12 modules and 79% (169/214) completed 10 modules or more. In all, 2 participants (1%) did not complete any modules, but did complete the assessments. Participants completed 9 of 18 (50%) available activities in the program on average; the number of activities completed ranged from 0 to 18. Several participants completed the same activity on multiple occasions, as allowed by the program, with the total number of activities completed ranging from 0 to 44 (mean 11.9, SD 9.1). Participants logged into the program an average of 18.7 times (SD 8.3, range 1-65). The mean total time spent in the program was 318.3 minutes (SD 204.3, range 24.7-1221.7).

On average, participants completed 0.5 activities per log-in (range 0-7.57) and 0.6 modules (range 0-2.0) per each log-in. The average amount of time spent online per log-in was 17.3 minutes (SD 10.5), whereas the average time to complete a module was 33.19 minutes (SD 23.18). For the combined usage measure of activities and modules, the mean score was 19.47 (SD 7.49).

### Demographic Factors, Usage, and Outcome

#### Overview

In all, 94 (43.9%) of participants obtained clinically significant improvement during the study. There was no difference between those who obtained clinically significant improvement and those who did not in age, level of education, country of birth, language spoken at home, marital status, or baseline depression score.

Older age was associated with greater time spent online (ρ=0.27, *P*<.001), more log-ins (ρ=0.19, *P*=.01), and total number of activities completed (ρ=0.16, *P*=.02). Men completed more modules (χ^2^_1_ = 5.0, *P*=.03) than women did. There were no sex differences in the other basic usage measures or obtaining a clinically significant outcome (χ^2^_1_ = 1.6, *P*=.69). A significant correlation was found between baseline depression severity and number of modules completed (ρ=–0.141, *P*=.04) with people who were more depressed completing fewer modules. However, there was no significant association between baseline depression severity and other usage metrics. No relationships were found between the interactions of age, sex, and baseline depression severity with usage on outcome.

#### Usage Factors Associated With Clinically Significant Improvement in PHQ-9 Score

Associations between basic and composite usage measures and clinically significant improvement were examined (Table 2). Of the basic usage measures, there was no significant difference between those who obtained clinically significant change and those who did not in the number of modules completed, the number of log-ins to the program, or the proportion of the 18 potential activities completed. However, a significant difference was found in the total number of minutes spent in the program between those who obtained clinically significant change on the PHQ-9 (mean 351.1, SD 206.4) and those who did not (mean 292.6, SD 199.8; *t*_212_=2.09, *P*=.04). Likewise, a significant difference was found in the number of activities completed between those who obtained change (mean 13.5, SD 9.5) and those who did not (mean 10.7, SD 8.7; *t*_212_=2.33, *P*=.02).

Of the composite measures, a significant difference was found between those who obtained clinically significant change and those who did not in average number of activities completed per log-in (mean difference 0.20, range 0.07-0.33; *t*_212_=3.02, *P*=.01) and average time spent online per log-in (mean difference 3.26 minutes, range 0.88-5.63; *t*_212_=2.71, *P*=.01). No other composite usage metrics were associated with significant outcomes.

A total of 214 cases were analyzed in a binary logistic regression model using the backwards likelihood ratio method. In total, 6 usage variables were associated with outcome in the univariate analyses at the *P*<.20 level as prespecified for inclusion in the model, but because of high autocorrelations, total number of activities completed and proportion of activities completed were removed. The combined measures were retained because they were considered a priori to provide a better reflection of use over time rather than the simple quantification of use.

The remaining usage measures (average minutes per log-in, average number of activities completed per log-in, and total time spent in the program) were entered into the model with sex and age. In the parsimonious model, total time spent online in the program, time spent online per log-in, activities completed per log-in, and the combined modules-activities measure remained in the model. Of these, only the number of activities completed per log-in was statistically associated with a clinically significant improvement (see Table 3.).

According to the Nagelkerke *R* ^*2*^ statistic, the variance in the outcome predicted by this model was 7.4%. The likelihood of the model predicting whether or not the individual would obtain clinically significant change or not was 61.2%. A further regression was modeled using the variables excluded based on autocorrelations as sensitivity analysis. This yielded similar results with only activities completed per log-in being found to contribute significantly to the final model.

To examine the linearity of the relationship between usage and outcome, the linear model of clinically significant change and significant usage metrics included in the linear regression were compared with logarithmic and quadratic curve estimation. Significant curve estimations were found for the 4 usage variables included in the final step of the analysis, except for the combined activities-modules metric (see Table 4), although they did not significantly outperform the linear model in any case.

**Table 2:** Univariate associations of usage metrics of E-couch with clinically significant change in depression in CREDO.

Variable	Overall sample, mean (SD)	Clinically significant improvement, mean (SD)^a^		Difference (95% CI)	*P* value	Effect size (Cohen’s *d*)
		Yes (n=94)	No (n=120)			
Proportion of 12 modules completed	0.9 (0.3)	0.9 (0.3)	0.9 (0.3)	0.03 (–0.05, 0.11)	.42^c^	0.11
Proportion of all 18 activities completed	0.5 (0.3)	0.6 (0.3)	0.5 (0.3)	0.07 (–0.20, 2.70)	.09^b^	0.23
Total number of activities completed (range 0-44)	11.9 (9.1)	13.6 (9.5)	10.8 (8.7)	2.89 (0.44, 5.34)	.02^c^	0.32
Number of program log-ins (range 1-65)	18.7 (8.3)	18.9 (7.2)	18.6 (9.1)	0.23 (–2.04, 2.49)	.84^b^	0.03
Total number of minutes spent online in program	318.3 (204.3)	351.1 (206.4)	292.6 (199.8)	58.42 (3.34, 113.47)	.04^b^	0.29
Average number of activities completed per log-in (range 0-2.4)	0.6 (0.5)	0.8 (0.4)	0.6 (0.5)	0.20 (0.07, 0.33)	.04^b^	0.46
Number of modules completed per log-in (range 0-2.0)	0.6 (0.3)	0.6 (0.3)	0.6 (0.2)	–0.01 (–0.08, 0.06)	.73^b^	0.06
Average number of minutes online per log-in (range 1.6-63.8)	17.3 (8.9)	19.1 (10.4)	15.9 (7.2)	3.26 (0.88, 5.63)	.01^b^	0.37
Average number of minutes online per module (range 5.6-165.8)	33.2 (23.8)	35.3 (22.9)	31.5 (24.5)	3.80 (2.69, 10.30)	.25^b^	0.16
Combined measured (range 0.0-30.0)	19.5 (7.5)	20.4 (7.0)	18.8 (7.8)	1.595 (–0.43, 3.62)	.12^b^	0.21

^a^Defined as a reduction of 5 points or more on the PHQ-9.

^b^ *t* test analysis.

^c^Mann-Whitney *U* test.

^d^Combined measure of number of compulsory activities completed (of possible 18) and number of modules completed (of possible 12) with scores ranging from 0-30.

**Table 3:** Final step in the binary logistic regression model using the enter ratio and adjusted for age and gender examining the relationship of usage measures to obtaining clinically significant change.

Usage variable	B	SE	Wald χ^2^	*P*	Exp(B)	95% CI
Time spent online in minutes	0.00	0.00	0.88	.35	1.00	1.00-1.00
Time spent online per log-in	0.01	0.03	0.08	.78	1.01	0.96-1.06
Activities completed per log-in	1.04	0.51	4.21	.04	2.82	1.05-7.59
Combined modules and activities measure	–0.04	0.03	1.24	.27	0.96	0.90-1.03
Constant	–0.86	0.30	8.13	<.001	0.42	

**Table 4:** Comparison of linear, logarithmic, and quadratic models for usage variables included in the linear regression.

Usage variable and model		Unstandardized coefficients		Standardized coefficients	*F* (df)	*P*	Adjusted *R* ^*2*^
		B	SE	β			
**Activities completed per log-in**							
	Linear	0.217	0.070	0.208	9.61 (1,212)	.002	0.039
	Logarithmic	0.105	0.040	0.178	6.98 (1,212)	.009	0.027
	Quadratic	0.023	0.101	0.044	4.81 (2,211)	.009	0.035
**Total time spent online**							
	Linear	0.000	0.000	0.142	4.38 (1,212)	.04	0.016
	Logarithmic	0.114	0.047	0.163	5.79 (2,212)	.02	0.022
	Quadratic	0.001	0.000	0.418	3.23 (2,211)	.04	0.020
**Total time spent online per log-in**							
	Linear	0.010	0.004	0.183	7.33 (1,212)	.007	0.029
	Logarithmic	0.146	0.064	0.155	5.21 (1,212)	.02	0.019
	Quadratic	0.001	0.011	0.022	4.04 (2,211)	.02	0.028
**Combined activities-modules metric**							
	Linear	0.007	0.005	0.106	1.22 (1,212)	.12	0.007
	Logarithmic	0.084	0.055	0.104	1.15 (1,212)	.28	0.001
	Quadratic	0.011	0.019	0.169	1.23 (1,211)	.30	0.002

#### Usage Groups and Outcome for Persisters

**Figure 1:**
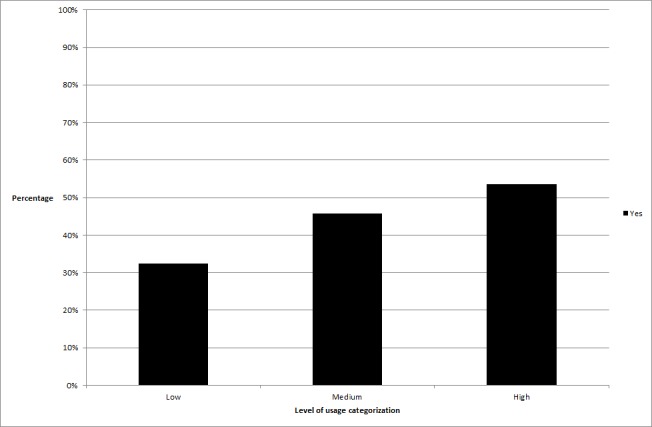
The difference in percentage of participants achieving clinically significant change across usage groups relating to total time spent online in the program.

**Figure 2:**
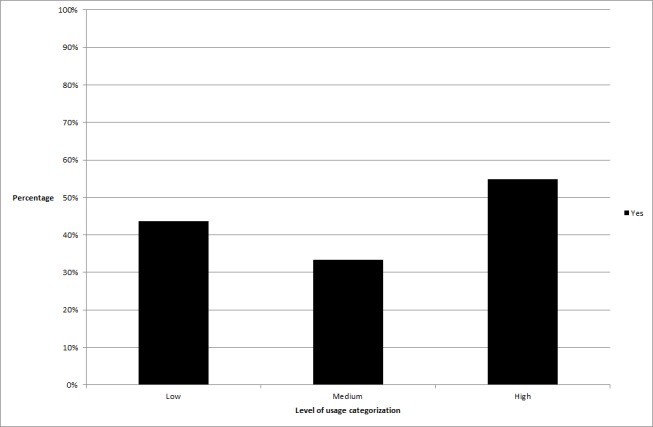
The difference in percentage of participants achieving clinically significant change across usage groups relating to time spent online per log-in.

**Figure 3:**
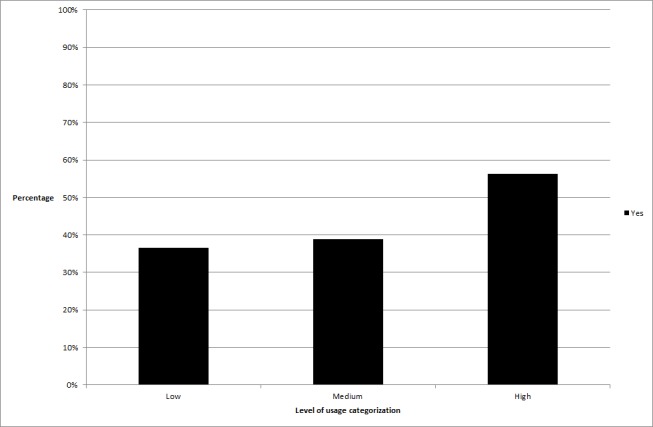
The difference in percentage of participants achieving clinically significant change across usage groups relating to activities completed per log-in.

Patterns of usage were also explored by trichotomizing usage metrics using tertiles of low, medium, and high users. When exploring this categorization against obtaining clinically significant change, significant relationships were found between outcome and time spent online (χ^2^_2_ =6.6, *P*=.04), time spent online per log-in (χ^2^_2_ =6.8, *P*=.03), and activities completed per log-in (χ^2^_2_ =6.7, *P*=.04). In the time spent online variable, significantly more high users obtained clinically significant change than low users (high users obtaining change = 53.5%, low users= 32.4%, *P*=.01), in time spent online per log-in, more high users obtained change than medium users (high users obtaining change = 54.9%, medium users= 33.3%, *P*=.01), and in activities completed per log-in, significantly more high users obtained change than low users (high users obtaining change = 56.3%, low users = 36.6%, *P*=.02) or medium users (medium users= 38.9%, *P*=.04). See Figures 1-3 for graphical representations of these findings.

### Sensitivity Analysis

A sensitivity analysis was completed using the continuous variable of PHQ-9 change score. This allowed for slightly increased power and the inclusion of more variables into the analysis. However, when a regression analysis was completed, activities completed per log-in remained the only significant predictor of outcome. Curve estimation did not meet significance for activities completed per log-in (logarithmic curve estimation: *P*=.06; quadratic curve estimation: *P*=.09) and time spent online per log-in (logarithmic: *P*=.09). Similar to clinically significant change, tertile splits found a significant difference between high and low/medium level users for time spent online per log-in and for activities completed per log-in.

## Discussion

### Principal Findings

Current schools of thought regarding the role of usage in Web-based interventions tend to draw on literature from other treatment paradigms with the assumption that benefit obtained from the program is proportionate to the level of program use. This study found relationships between only a few objectively measured usage variables and clinically significant improvement in participants who persisted with the iCBT program. Of the 4 usage variables that were included in the linear regression model, only the number of activities completed per log-in independently predicted outcome in the final regression model. Further, the significance of nonlinear models for several of the usage variables indicates that the relationship between use and outcome may not be as simple as a linear relationship. Instead, it supports a view that the benefits of use may occur after following a high level of activity during each engagement with the program, not necessarily as a result of ongoing longer-term engagement with the program, and that the number of modules completed in the program is a poor indicator of benefit obtained. Likewise, the analysis of levels of usage indicates that although high program users generally do better, medium users do not necessarily benefit more than low users.

Across analyses, it appears that those participants who were more actively engaged in the program (completing more activities each time they logged in and spending more time in the program with each log-in) were most likely to benefit from the program. These findings are not dissimilar to face-to-face CBT, in which it is the ongoing completion of homework activities across therapy sessions that best predicts outcome [35]. Thus, this indicates that users who are more actively engaged in their treatment may do better.

The activities completed per log-in metric accounted for very little of the variance in the outcome. The inability of some other metrics to predict outcome may reflect limited variability, particularly concerning modules completed, a metric that has been associated with outcome previously [16]. Within this study, the level of module completion (all modules completed by 62.1% of participants) and number of log-ins (a mean of 18 when 12 would have been required to complete the program) indicates a relatively high usage. The other metrics of usage showed greater variability and were more likely to be associated with outcome, with the variable with the greatest variance (activities completed per log-in) being the most strongly related to outcome.

As expected, participants who did not persist with the trial provided outcome data with lower levels of usage. This is likely to reflect early dropout and not being exposed to the content; therefore, they were unable to be more adherent to the program overall. People who were older appeared to adhere more, consistent with other studies [36]. However, the longer time spent online in the older age groups may not actually be a good measure of use because it may reflect less familiarity with using the computer or a slower cognitive processing speed rather than indicating something that may influence outcome. It was also found that men completed more modules than women did, somewhat contrary to previous studies [36,37], although a recent systematic review failed to consistently find a relationship between demographic variables and usage [37] indicating that other factors, such as patient beliefs and personal motivations, may also influence usage [38]. Despite sex and age being found to be associated with the number of activities completed, no interaction was found between these demographics, usage, and outcome. Likewise, no interaction was found between baseline depression severity, number of modules completed, and outcome. Therefore, this supports the view that program usage has a greater impact on outcome than demographics.

The lack of any strong predictive relationship between the usage metrics identified here and outcome may challenge the traditional view of a dose-response relationship relating to outcome. The high rates of usage in this study may have meant that many participants reached a dose-response plateau where they had been exposed to an appropriate level of the program and were unlikely to obtain further benefits from additional exposure. This model indicates that patients may reach therapy saturation at certain levels of use and would likely obtain the effects of the program early on. If this were the case, we would expect that outcome gains would be obtained with medium usage and then be maintained as patients persisted with the intervention. Such an effect has been seen in longer Web-based interventions [15]; for example, Christensen et al [39] found no further improvement in symptoms between 4 and 5 modules. Conversely, of those usage metrics that were associated with outcome here, medium users appeared to derive minimal if any benefit compared to low users and it was the high users that benefited, implying a difference between modules delivered and adherence.

The high rates of use may reflect this analysis only selecting participants that persisted with the study, whereas other usage-outcome association studies have utilized the last observation carried forward (LOCF) technique. Although retaining only those who had persisted may have biased the analysis to more adherent people, using the LOCF technique conflates the measurement of persistence (the number of people who complete the program) which, in turn, leads to these participants also appearing to have poor usage (because only a small proportion of the program completed). Additionally, LOCF may also underestimate the overall effectiveness of the intervention because some studies have reported that people who notice an improvement in their symptoms drop out [37,40], but a LOCF approach would assume no improvement. Additionally, the analysis could have included more complex and potentially more accurate methods for handling missing data, such as multiple imputations or mixed models with maximum likelihood estimation. Given that missing outcome data was likely to occur in cases with low usage rates (because of not completing the program and not providing outcome data), and that the aim of the analysis was to explore this relationship, complete case analysis was preferred.

The univariate associations between usage and outcome found in this analysis are consistent with our recent review which indicated that, of the online intervention studies which reported usage, most (31/33 studies) found a positive relationship between usage variables (34/37 variables) and outcome [16]. However, when further analysis is completed, such as within the present paper, the ability of these variables to predict improvement in the form of clinically significant change is limited. As such, these findings coupled with the curve estimations in this analysis may challenge the perception of the linearity of the relationship between usage and outcome. This implies that it is not the exposure to the material alone that improves outcome, as evidenced by the lack of association between basic usage metrics such as modules completed, but the gradual exposure to and active engagement with the material over time, as evidenced by the strong relationship of composite variables. Given this, we can conclude that concentrated use of the program (eg, completing multiple modules per log-in) or passive exposure to material (as measured by modules completed) may not be as useful as regular shorter periods of use with higher levels of activity in each of these log-ins.

### Future Direction

A number of recommendations based on these findings can be made. The finding that those who completed a high number of activities per log-in achieved a greater benefit than those who undertook few, and that a medium activity per log-in count conferred no more benefit than a low activity per log-in count indicates that maximizing usage behavior online may improve outcomes. One way of doing this is ensuring that more activities are included with each module, thereby encouraging users to be more engaged with their treatment. These activities could include the use of activities related to the therapeutic modality of the program or multiple choice quizzes to assess learning with reference back to sections containing material related to incorrect answers.

Programs that limit program exposure at each log-in to allow adequate time for learning, the completion of activities, and skills implementation to occur may also be beneficial as well as incorporating a “hook” to encourage users to return the following week [38]. This directive and potentially restrictive nature of the intervention needs to be balanced with user perception of freedom within the program to encourage ongoing engagement [41].

Providing education and setting early expectations about what users need to do to achieve benefit from the program (ie, being more active while online) may be a helpful approach to improving program outcomes. This would include emphasizing that users are more likely to obtain benefit if they are more active and complete activities when they become available. Likewise, encouraging users to complete the activities on multiple occasions, particularly when waiting for the next module to become available, may also improve outcomes. However, definitive conclusion about the usefulness of these strategies is beyond the scope of this analysis and would benefit from further research.

A further opportunity is to measure usage and program benefits throughout the course of the intervention to determine at what point users reach their therapy saturation and obtain little if any program gains after this time point. Conversely, such monitoring may indicate that a certain level of usage is required to obtain a benefit. Ensuring users are actively engaging in the program is likely to require frequent monitoring, which in itself can influence the outcome of the intervention, and the use of measures with good test-retest properties. Future programs utilizing this design will also need to consider the burden of intensive monitoring on users and the potential for this to increase the propensity to drop out from the study. Electronic measurement automation may provide a way to reduce monitoring burden.

Previous research has found that although usage of program components was related to early improvements, it was the completion of homework exercises that was correlated with long-term improvements [42]. Given this, developing and using a measure designed to capture real world implementation of online learning and completion of offline homework activities may be key in better understanding how program use may impact outcome. This may be as simple as asking users if they have completed their homework tasks or providing details of how they have implemented the previous module’s learnings, much like feedback occurs in current psychotherapy. However, reporting is likely to be prone to self-report bias and may only provide a crude estimate until more sophisticated tools are developed. Standardizing the assessment of usage across trials and programs would be of huge benefit in understanding these processes [21] and suggests a role for multinational nongovernment organizations and developers groups, such as the International Society for Research on Internet Interventions, in this process. However, until a consensus about the best way to measure and define usage is reached, it is difficult to implement standardization throughout trials.

### Limitations

As mentioned previously, the inclusion of only those participants who provided outcome data is likely to have limited the generalizability of the results. However, it is unclear what effect that this may have had on the analysis and how to best manage this. Using the LOCF may actually fail to give an accurate picture of the progress of these participants. This is largely because the LOCF method assumes no progress in this group. Although research has indicated that obtaining benefits may be key in helping people to persist with an intervention [38], it may also contribute to users ceasing to use the intervention because they believe that they no longer require assistance. Given this, generalizing assumptions about usage to those who fail to persist with interventions should be done with caution. Additionally, usage research may benefit from using more sophisticated analytic approaches (eg, latent class modeling [43,44] or gaussian mixture models [45]) to assess whether there are groups who are more likely to respond and if these groups differ in usage. Growth modeling of outcomes and usage would be better able to analyze the usage-outcome association.

In addition, the specific inclusion criteria of this trial and the unique nature of each intervention likely limits the generalizability of the results of this analysis to other populations. However, recent findings have indicated that this may be less of a factor than initially thought, and that the sampling bias related to trials may not actually limit the ability of trial results to be generalized based on demographic factors [46].

Finally, despite that the data were drawn from a RCT, this substudy was observational in nature and no manipulation of variables related to usage occurred. Given this, the ability to imply causation is limited. Future research needs to explore the manipulation of these variables, such as controlling number of log-ins to determine if unlimited access affects outcomes, exploring usage and outcome in single log-in sites, or limiting the amount of activities or modules that can be completed per log-in to future test the hypotheses drawn from the findings of this study.

### Conclusions

Future research would benefit from exploration of the relationship between usage metrics and outcome to further investigate the nature of this relationship. Although this analysis found only 1 metric was predictive of outcome, this finding is limited by the context of this study. Future research needs to continue to explore this research in trials and naturalistic implementation of Web-based interventions to determine if this is the case.

## Abbreviations

CBT: cognitive behavior therapy
CREDO: Cardiovascular Risk E-couch Depression Outcome
CVD: cardiovascular disease
iCBT: Internet cognitive behavior therapy
IPT: interpersonal psychotherapy
LOCF: last observation carried forward
RCT: randomized controlled trial

## Multimedia Appendix 1

Screenshot showing an example of psychoeducation about the link between thoughts and moods.

## Multimedia Appendix 2

Screenshot showing psychoeducation teaching the user about cognitive distortions.

## Multimedia Appendix 3

Screenshot showing an example of an activity that teaches users about cognitive restructuring.

## Multimedia Appendix 4

Screenshot showing an example of an activity from the interpersonal therapy component of E-couch.
