# Supplementary figures and images for: Rethinking the Dose-Response Relationship Between Usage and Outcome in an Online Intervention for Depression: Randomized Controlled Trial
Source: J Med Internet Res. 2013 Oct 17;15(10):e231. doi: 10.2196/jmir.2771 (PMC3806549; doi:10.2196/jmir.2771)

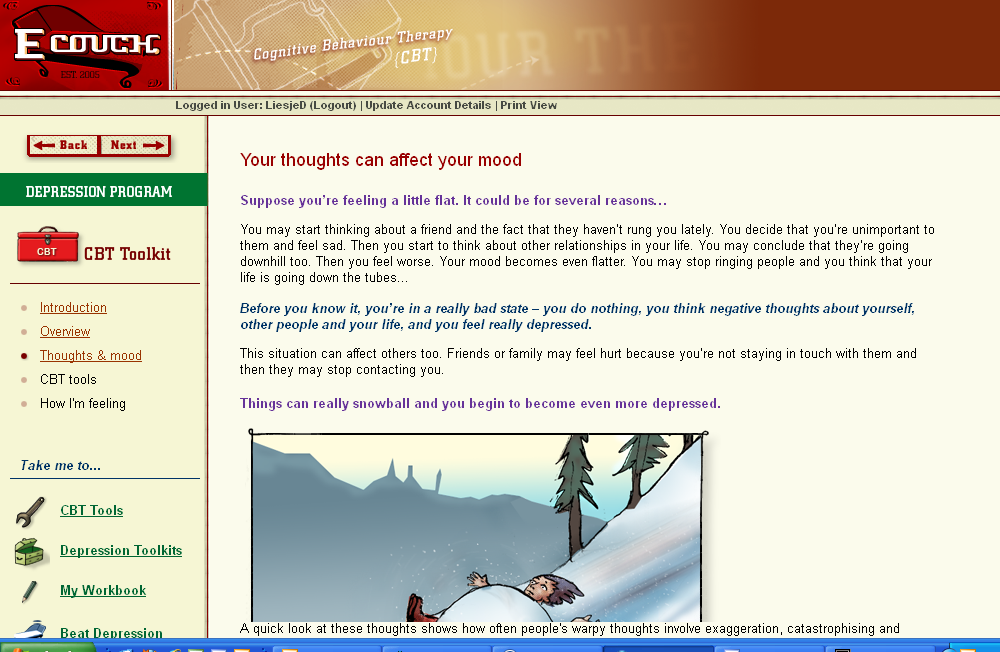

Supplement: Supplementary file 1 [file jmir_v15i10e231_app1.png]

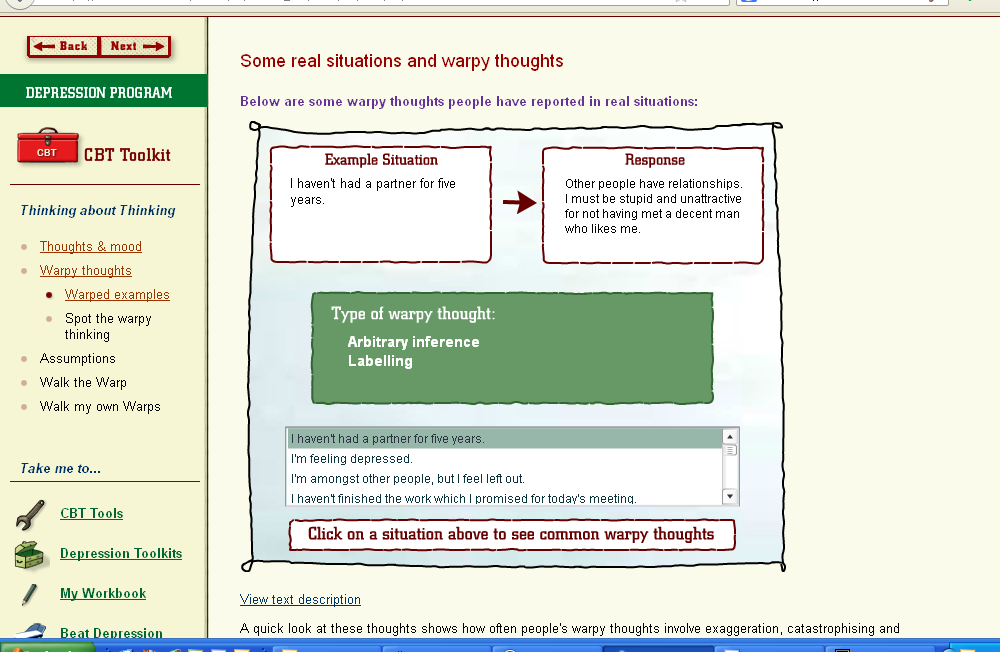

Supplement: Supplementary file 2 [file jmir_v15i10e231_app2.png]

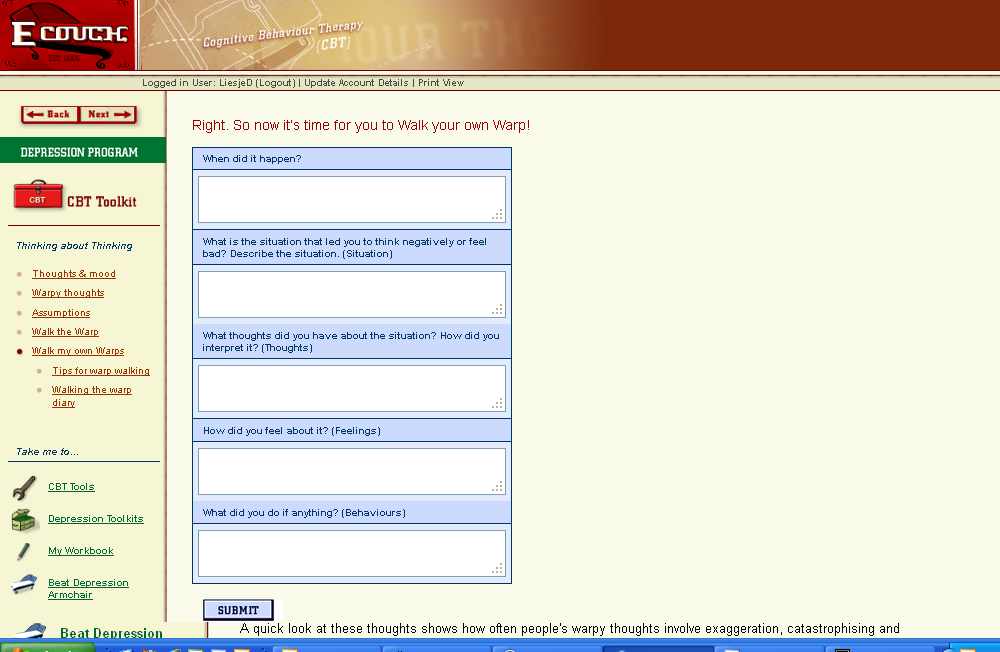

Supplement: Supplementary file 3 [file jmir_v15i10e231_app3.png]

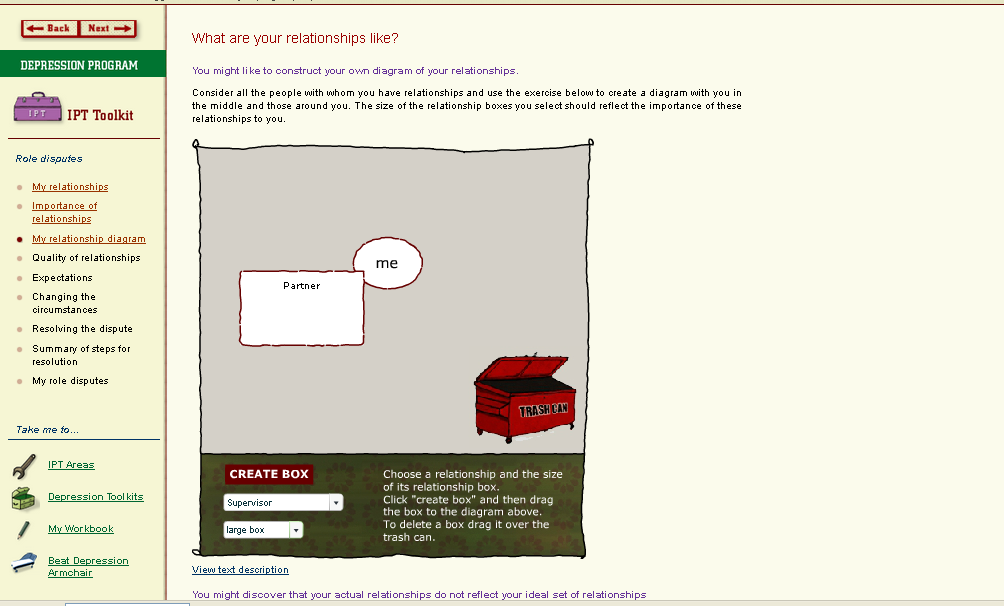

Supplement: Supplementary file 4 [file jmir_v15i10e231_app4.png]
